# The reproducibility of measurements of intramuscular magnesium concentrations and muscle oxidative capacity using ^31^P MRS

**DOI:** 10.1186/1476-5918-8-5

**Published:** 2009-12-15

**Authors:** Kevin K McCully, Tiffany N Turner, Jason Langley, Qun Zhao

**Affiliations:** 1 Department of Kinesiology, University of Georgia, Athens, GA, USA; 2 Department of Physics and Astronomy & BioImaging Research Center, University of Georgia, Athens, GA, USA

## Abstract

^31^P magnetic resonance spectroscopy (^31^P MRS) has been used to measure intramuscular magnesium concentrations and muscle metabolism. Abnormal intramuscular magnesium has been reported in several patient populations with suspected metabolic disorders. The purpose of this study was to evaluate our ability to measure intramuscular magnesium and muscle metabolism in the quadriceps muscles of healthy subjects, and to test whether these measurements were influenced by prior exercise. Twelve normal, healthy male volunteers were tested in a 3 Tesla magnet on four separate days. Resting [Mg^2+^] was calculated from the heights and frequency shifts of the phosphate, phosphocreatine and ATP peaks. Phosphocreatine (PCr) recovery kinetics were measured after 30-39 second bouts of isometric exercise. Thirty minutes prior to the 3^rd ^test session the subjects completed a 2 hour treadmill walk at 40-60% of heart rate reserve. Resting [Mg^2+^] averaged 0.388 mM and had an interclass correlation coefficient between days (ICC) of 0.352. The mean end exercise PCr was 47.6% and the mean end exercise pH was 6.97. PCr recovery averaged 39 seconds (p = 0.892) and had an ICC of 0.819. Prior long duration exercise did not produce significant alterations in either PCr recovery kinetics or intracellular magnesium levels (p = 0.440). In conclusion, the reproducibility of Resting [Mg^2+^] was less than that of PCr recovery measurements, and may reflect the sensitivity of these measurements to phasing errors. In addition, prior exercise is unlikely to alter measurements of resting metabolites or muscle metabolism suggesting that rigorous control of physical activity prior to metabolic testing is unnecessary.

## Background

Magnesium is a relatively abundant element in the body that is important in a number of metabolic reactions[1]. A number of factors may change magnesium levels in the body. Physical activity may deplete magnesium concentrations which could lead to reduced exercise capacity [2-4]. In addition magnesium concentrations have been linked to changes in immune function and disease [5]. Many of the studies on magnesium have been made on serum and RBC magnesium concentrations[6,7], but other studies have suggested that magnesium levels in other tissues may also be important[8].

Intracellular magnesium concentrations [Mg^2+^] have been measured non-invasively in brain and skeletal muscles using ^31^P MRS[9,10]. This method is based on the frequency shift of the *β*-ATP peak caused by physiological concentrations of [Mg^2+^]. Alterations in either blood or muscle [Mg^2+^] have been reported in various patient population groups [10-13]. Several previous studies have suggested that patients with enhanced fatigue have altered intramuscular magnesium levels [14,15]. While these studies appear promising, relatively few other investigators have evaluated potential changes in [Mg^2+^].

A potential limitation to using ^31^P MRS measurements to calculate changes in [Mg^2+^] is that this method depends on relatively small frequency shifts in the *β*-ATP peak, which could potentially increase the variability of the measurement. It is also not clear how physical activity might influence [Mg^2+^] measurements. Strenuous exercise that alters immune function might be expected to alter [Mg^2+^], but it is less clear how relatively normal levels of physical activity might influence measurements of [Mg^2+^]. Similarly, it is not clear how prior physical activity influences measurements of muscle oxidative metabolism. Strenuous exercise which results in muscle injury or ATP loss might impair muscle metabolism as measured by the ratio of inorganic phosphate to phosphocreatine (PCr) during steady level exercise[16,17]. However, short duration acute exercise did not alter the rate of PCr resynthesis (a marker of oxidative metabolism)[18]. Subjects undergoing ^31^P MRS testing might be active during the day prior to testing or even have to walk considerable distances to reach the testing facility. It is not clear what kind of recommendations concerning prior physical activity are needed before ^31^P MRS testing can be performed.

The purpose of this study was to: 1) determine the reproducibility of measurements of [Mg^2+^] and muscle oxidative metabolism using ^31^P MRS, and 2) determine if [Mg^2+^] and PCr recovery kinetics are influenced by prior exercise. It was hypothesized that the reproducibility of [Mg^2+^] would be similar to that of PCr recovery kinetics, and that walking exercise of long duration prior to ^31^P MRS testing would change [Mg^2+^] and slow PCr recovery kinetics.

## Methods

A total of 12 healthy, college aged males (mean age = 22 ± 1.9 yrs) participated in this study (Table 1). All subjects participated in physical activity at least twice a week. The study was conducted with the approval of the Institutional Review Board at the University of Georgia and all subjects provided written informed consent.

**Table 1:** Subject characteristics

	Subject characteristics			Physical activity		
	Age	Height	Weight	Min/week		
	Years	Cm	Kg	Light	Moderate	Heavy
Mean	22	179	82	131	76	30
SD	1.9	3.7	33.1	78.5	42.3	31.8

n = 12, all male.

**Figure 1:**
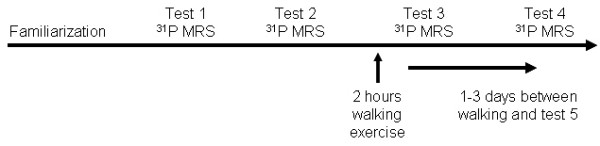
**Study time line**. The subjects were tested on five separate days. The first day was used as a training session outside the magnet. The remaining four days involved testing in the magnet.

This study used a one group with repeated measures experimental design. Each subject was tested on four separate days, plus one familiarization testing session (Figure 1). Each test day was separated by 1-30 days. The testing sessions consisted of ^31^P MRS measurements of resting [Mg^2+^] and muscle metabolism. Prior to test session three, the subjects performed inclined treadmill walking for two hours. Test session four was performed one to three days post exercise. Each subject reported no physical activity twenty-four hours prior to testing other than the exercise performed in this study.

### Familiarization session

A familiarization session was used to instruct the subjects how to perform the maximal isometric contractions (MVIC) in a supine position for 39 seconds. Subjects were positioned supine on a table and instructed to maximally contract the quadriceps muscle. Each subject performed two 20 second maximal contractions and two 40 second maximal contractions with approximately five minutes between contractions. To encourage maximal muscle activation, EMG signals were recorded from the vastus lateralis muscle and the subjects were allowed to view the signals. To predict if the contraction was vigorous enough to deplete PCr, the decline in oxygen saturation in the vastus lateralis muscle was measured during the maximal isometric contractions using continuous wavelength near-infrared spectroscopy (NIRS) [19].

### Intramuscular magnesium

Subjects were placed in a 3 Tesla whole body magnet (GE Healthcare, Waukesha, WI). A ^1^H and ^31^P dual radio-frequency (RF) surface coil (Clinical MR Solutions, Brookfield, WI.) was placed over the vastus lateralis of the subject's right leg. The size of the ^31^P coil was 13 cm × 13 cm with an overlapping ^1^H coil (two loops, side by side, 20 cm diameter). Manual shimming on ^1^H was applied to get a better signal-to-noise ratio (SNR) and less spectrum distortion, after an auto-shimming by a pre-scan sequence. A free induction decay (FID) chemical shift imaging (CSI) pulse sequence was applied to acquire the ^31^P spectrum. The scan parameters were: repetition time (TR) = 3000 ms, field-of-view (FOV) = 18 cm, slice thickness = 100 mm, number of excitation (NEX) = 1, rfpulse = hard. Resting spectra were acquired every 3 seconds until 120 scans were taken. The resulting spectra were zero filled (from 2048 to 6144 points) phased and averaged in a custom analysis program (Winspa, Ronald Meyer, Michigan State University). The area under the curve for each peak (*Pi, PDE, PCr, α ATP, β-ATP, and γ-ATP*) was determined using integration. Magnesium and pH were calculated using the following equations:

where *shift*_*β*-*ATP *_and *shift*_*pi *_represent a shift of the peak *Pi *and *β*-*ATP *in the spectrum, respectively.

### Phosphocreatine recovery kinetics

Muscle metabolism was measured using rate of recovery of PCr after exercise [15]. After resting measurements of [Mg^2+^] and ATP were taken, the subject performed a 30-39 second duration MVIC to deplete PCr. The subject was then instructed to remain as still as possible while recovery data was collected for approximately four minutes. This procedure was repeated two or three times. Phosphocreatine peaks were determined from peak heights from individual spectra using a custom Matlab (The Mathworks, Natick, MA.) routine. PCr peak heights during recovery after exercise were fit to an exponential curve:

where *A, B*, and *T*_*c *_are fitting parameters describing the PCr depletion.

### Exercise protocol

Incline treadmill walking was performed for two hours where the subjects reached a target work level that was between 40-60% percent of their heart rate reserve. Periodically during the walking, speed/grade variations, EMG activity, heart rate and perceived exertion were collected. EMG activity during walking was normalized to a MVIC performed prior to walking. Speed was adjusted between 3-5 mph and grade between 5-8% during the walking to ensure the testing remained at a consistent exercise intensity. Subjects were allowed to drink 32 ounces of water or a sports beverage while walking.

### Statistical analysis

All values are reported as means ± standard deviation. Repeated measures ANOVA and Reliability measures (SPSS) were conducted to evaluate the variation between and within days. A linear regression was used to evaluate whether oxygen saturation as a percentage of exercise could predict a person's end exercise PCr. Analyses were conducted with statistical significance accepted at *α *< 0.05. ICC values were calculated from a one way ANOVA, where the values were for each of the first two days. R = (mean square between - mean square within)/(mean square between + mean square within). The COV% was calculated as the standard deviation of the value for the first two days divided by the average value times 100%.

## Results

### Isometric contractions

All subjects in the study were able to consistently perform maximal MVIC contractions of their right leg during the familiarization session. This was evidenced by consistently high EMG signals and consistent depletion of oxygen saturation as measured by NIRS (end exercise oxygen saturations of 47 ± 20% compared to a resting value of 83 ± 4.6%).

### ^31^P MRS measurements

**Figure 2:**
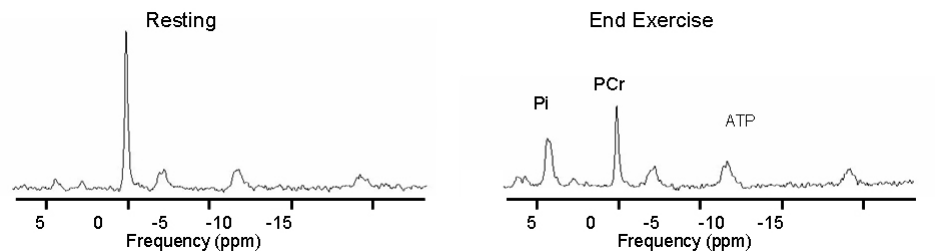
**Representative resting and end exercise spectra from one subject**. Each spectra is one average with a repetition time of 3 seconds.

Representative ^31^P spectra are shown in Figure 2. The mean resting phosphocreatine signal to noise ratio for all subjects was 47:1. A signal to noise ratio for phosphocreatine of 7:1 for individual spectra was set as exclusion criteria. Based on exclusion criteria for Mg and ATP concentration, all twelve subjects were analyzed. Table 2 shows the average values for the phosphorous peaks and for pH during the different trials both at rest and at the end of the in magnet exercise protocol.

**Table 2:** Changes in muscle metabolites pre/post exercise

		Rest Pi/PCr	Rest PCr/ATP	Rest pH	End exercise PCr (%)	End exercise pH
Pre 1	Mean	**0.089**	**4.24**	**7.07**	**46.1**	**6.94**
	SD	0.029	0.66	0.03	11.6	0.07
Pre 2	Mean	**0.092**	**4.68**	**7.06**	**52.0**	**6.97**
	SD	0.012	0.98	0.02	14.0	0.10
Post 1	Mean	**0.080**	**5.14**	**7.06**	**48.9**	**6.98**
	SD	0.017	0.98	0.02	14.0	0.10
Post 2	Mean	**0.092**	**5.12**	**7.05**	**43.5**	**6.98**
	SD	0.023	1.08	0.02	7.8	0.08
ICC pre 1&2		**0.13**	**0.79**	**.021**	**0.87**	**.064**
COV% pre 1&2		**15.1**	**8.60**	**0.21**	**11.1**	**0.79**

### Intramuscular magnesium concentrations

Magnesium concentrations during the four test sessions are shown in Table 3. Mauchly's W was significant (W = 0.030, p < 0.001), indicating unequal variance between testing sessions. This was a result of the two outlying values (0.57 and 0.24 mM) occurring in the first test results. These differences were consistent when the data was analyzed with two independent observers. After using a Greenhouse-Geisser correction for unequal variance, there was no statistically significant differences in [Mg^2+^] between the four test sessions (p = 0.440). This did not support the hypothesis that [Mg^2+^] would change as a result of the exercise session.

**Table 3:** [Mg^+2^] changes pre/post exercise

	Pre 1	Pre 2	30-60 Minutes Post exercise	1-3 Days Post exercise
Mean	0.414	0.376	0.386	0.375
SD	0.151	0.034	0.035	0.037
				
ICC b/t pre 1&2	0.352			
COV% b/t pre 1&2	7.91			

### Muscle metabolism measured with PCr recovery kinetics

**Figure 3:**
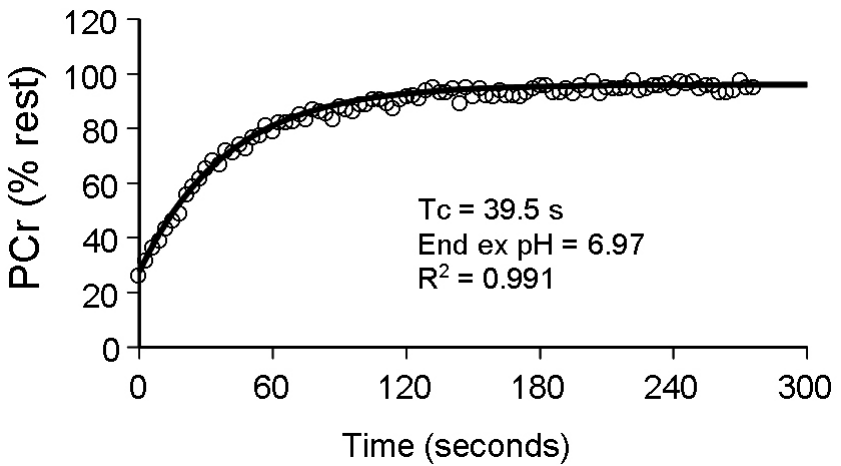
**Representative PCr recovery curve after isometric exercise**. Each data point is the peak PCr value for a single average spectra.

Figure 3 shows a representative recovery curve for phosphocreatine after exercise. PCr recovery data were used in the analysis if the exercise resulted in a depletion of at least 20% of PCr, and the curve fit of the recovery of PCr had an r^2 ^value above 0.6. Eleven of the twelve subjects had data that fit these criteria for all time points. PCr recovery time constants are shown in Table 4. There were no statistically significant differences in the PCr recovery kinetics (F = 0.206, p = 0.892, df = 3) between the four test days using ANOVA. This did not support the hypothesis that PCr recovery kinetics would change as a result of the exercise session. The correlation coefficient for PCr Tc values and [Mg^2+^] values averaged over the first two test sessions as low, R^2 ^< 0.02.

**Table 4:** Changes in PCr Tc to recovery pre/post exercise

	Pre 1 (Seconds)	Pre 2 (Seconds)	30-60 Minutes Post exercise (Seconds)	1-3 Days Post exercise (Seconds)
Mean	39.9	39.1	37.5	38.7
SD	12.7	16.9	14.0	12.4
				
ICC b/t pre 1&2	0.819			
COV% b/t pre 1&2	18.4			
ICC within pre 1	0.928			
COV% within pre 1	8.43			

## Discussion

This study found that the reproducibility of resting [Mg^2+^] as measured by COV% (7.9%) to be consistent with although slightly higher than literature values of 6.1% [20] and 4.1% [21]. One reason for the slightly higher variability in our study was the presence of the highest (0.57 mM) and lowest (0.24 mM) [Mg^2+^] values in the first testing session. Without these high/low values, the mean and standard deviation of the first test session was similar to the other test days. We have no explanation for these outlying values, and separate analysis of all the data by a different investigator did not change the pattern of the results. Variability reported as ICC values were lower than what is expected for data that is considered to have adequate reproducibility (0.362, which is below 0.70). The COV% and ICC values illustrate what we think is an important aspect of the [Mg^2+^] measurements. The good COV% suggests that the [Mg^2+^] are reproducible. The low ICC values on the other hand suggest there is little difference between [Mg^2+^] values between healthy control subjects. Thus, [Mg^2+^] measurements might be useful for detecting differences in patient populations where [Mg^2+^] might be expected to change [10-15], but not for detecting differences within normal healthy populations.

The actual values for [Mg^2+^] were consistent between our current study (0.39 mM) and our previous study (0.37 mM) [13], despite using a different muscle (vastus lateralis versus the gastrocnemius muscle) and a different magnet/spectrometer system. These values are slightly higher than found in several other previous studies (0.31 and 0.32 mM) [12,22], although they are are lower than values (0.56 mM) from the calf by Ryschon et al. [20]. We used the same equation for determining [Mg^2+^] as did[12,22]. However, Ryschon et al. [20] used a different equation to determine [Mg^2+^], which used the frequency shifts between the ATP peaks[23] rather than between PCr and the beta ATP peak as in this study. Because the frequency shift between PCr and beta ATP was similar between our study (-15.85 to -16.03 ppm) and that of Ryschon et al. (-15.9 to -16.1 ppm) [20], it is most likely the equation used rather than measurement differences that explain the differences in concentrations found.

We found that measurements of intramuscular magnesium were not affected by prior exercise. Numerous studies have reported sweat, urinary, and blood magnesium changes with exercise [1,4,24]. Increases in urinary magnesium excretion [25] and decreases in serum magnesium levels [26] have been reported after running a marathon. Muscle magnesium may increase after exercise[1,27]. Changes in magnesium concentrations with exercise could be due to dehydration and redistribution of magnesium around the body, or be related to inflammatory responses related to exercise[28]. The exercise used in this study may not have activated the quadriceps muscles enough to directly influence muscle magnesium. This is because only a small amount of the quadriceps muscle is actually active during walking (we estimated this to be around 2% based on EMG signals). However, marathon running was shown to change serum magnesium concentrations. Our exercise protocol was two hours in duration, and while classified as a moderate intensity, was designed to produce a reasonable amount of stress on the body. It maybe that future studies will need to use exercise that activates the target muscle more, or a more strenuous exercise designed to produce a large immune response. Our results do suggest that it is not necessary to strictly control activity levels of subjects prior to making intramuscular magnesium measurements. Two hours of moderate intensity walking is more than most potential research subjects would perform prior to testing.

This study found no relationship between intracellular magnesium levels and muscle metabolism measured by PCr recovery kinetics. Previous studies have suggested that since magnesium in a primary stabilizing cofactor in ATP dependent reactions [1], changes in bound magnesium levels may alter the phosphorylation and dephosphorylation of mitochondrial ATP [9]. The phosphate from the mitochondrial ATP is used to rephosphorylate PCr; therefore, changes in this oxidative metabolism may be reflected through magnesium concentrations and/or phosphocreatine recovery kinetics. The finding that baseline measurements of [Mg^2+^] did not correlate with the PCr Tc, which did have an acceptable ICC values, support the previous discussion that magnesium measurements maybe more useful in detecting abnormal populations or conditions rather than differences between healthy younger subjects as tested in this study.

The reproducibility of our PCr recovery kinetics was similar to that reported in previous studies[29]. We found a COV% of 8% within a test session and 18% between test sessions. Walter et al [18] tested four people on two different days and found a k_pcr _COV% of 8-10% for varying workloads and durations in the calf muscle. Larson-Meyer et al. [30] found a COV% of 5.0 ± 2.9 s after testing the calf of eight female subjects one month apart. Unlike the [Mg^2+^] measurements, the ICC values for PCr recovery time constants were above 0.70, indicating a good ability to detect differences between normal healthy subjects.

The PCr recovery time constant was not influenced by two hours of vigorous walking. In previous studies, repeated bouts of short duration exercise did not alter PCr recovery kinetics [31], and strenuous exercise performed two days prior to the measurements did not alter PCr recovery in either normal controls or people with chronic fatigue syndrome [15]. Walter et al. [18] also measured PCr recovery kinetics in the gastrocnemius of eight male subjects, measured as V_max_, with and without a warm-up and did not find a significant difference in the times to recovery. Together these results suggest that both PCr recovery kinetics as well as intramuscular magnesium measurements are relatively insensitive to prior physical activity.

The PCr recovery measurements differed from previous studies in that we did not use an in magnet ergometer. The use of a MVIC is advantageous because it does not require a special in-magnetic ergometer. This type of exercise was considered unusual by the research subjects and thus we felt that a familiarization test session was needed. We did not find that oxygen saturation values measured during this test were useful in predicting which subjects would deplete the most PCr during the in-magnet tests. However the use of EMG signal did seem to have a small effect on the subjects' ability to activate their vastus lateralis muscles. We found that a MVIC of the quadriceps was sufficient to deplete PCr and generate a mono-exponential recovery curve. Measurements of PCr using ^31^P MRS have been shown to agree with measurements from biopsies [32], and the recovery rate of PCr has been correlated with biopsy measurements of mitochondrial enzymes[33]. Thus, we feel the use of isometric contractions to deplete PCr, with the subsequent measurement of PCr recovery rates is an acceptable method of measuring muscle metabolism.

## Conclusions

We found that measurements of [Mg^2+^] and PCr recovery kinetics to be reproducible, but in different ways. [Mg^2+^] have lower COV%, but also lower ICC indicating good reproducibility but poor ability to detect differences between healthy control subjects. PCr recovery kinetics showed less reproducibility with COV%, but with an adequate ability to detect differences between healthy control subjects as shown by high ICC values. Neither [Mg^2+^] or PCr recovery kinetics were influenced by 2 hours of vigorous walking exercise. This may reflect the relatively low activation of the vastus lateralis during vigorous walking. It does suggest that limiting subject walking activity prior to testing for [Mg^2+^] and PCr recovery kinetics is not necessary.

## Abbreviations

COV%: coefficient of variation (as a percentage); EMG: electromyography; ICC: intraclass correlation coefficients; Mg^2+^: magnesium; MVIC: maximum voluntary isometric contraction; ^31^P MRS: ^31^Phosphorous magnetic resonance spectroscopy; PCr: phosphocreatine.

## Competing interests

The authors declare that they have no competing interests.

## Authors' contributions

All authors read and approved the final manuscript. KM designed the study, supervised all aspects of the study, and performed all administrative tasks associated with the study, revised manuscript. TT recruited subjects, conducted the testing, performed initial data analysis, wrote initial draft of manuscript. JL assisted with ^31^P MRS data analysis, assisted with manuscript revision. QZ assisted with ^31^P MRS data analysis, assisted with data interpretation, assisted with manuscript revision.
